# Lipostatic Mechanisms Preserving Cerebellar Lipids in MPTP-Treated Mice: Focus on Membrane Microdomains and Lipid-Related Gene Expression

**DOI:** 10.3389/fnmol.2019.00093

**Published:** 2019-04-24

**Authors:** Mario Díaz, Ana Canerina Luis-Amaro, Deiene Rodriguez Barreto, Verónica Casañas-Sánchez, José A. Pérez, Raquel Marin

**Affiliations:** ^1^Departamento de Biología Animal, Edafología y Geología, Universidad de La Laguna, San Cristóbal de La Laguna, Spain; ^2^Unidad Asociada de Investigación ULL-CSIC, “Fisiología y Biofísica de la Membrana Celular en Patologías Neurodegenerativas y Tumorales”, San Cristóbal de La Laguna, Spain; ^3^Departamento de Ciencias Médicas Básicas, Universidad de La Laguna, San Cristóbal de La Laguna, Spain; ^4^Instituto Universitario de Enfermedades Tropicales y Salud Pública de Canarias, San Cristóbal de La Laguna, Spain; ^5^Departamento de Bioquímica, Microbiología, Biología Celular y Genética, Universidad de La Laguna, San Cristóbal de La Laguna, Spain

**Keywords:** cerebellum, Parkinson’s disease, membrane microdomains, membrane lipids, MPTP toxicity, lipid-related gene expression, dopamine transporter

## Abstract

The cerebellum is an essential component in the control of motor patterns. Despite dramatic alteration of basal ganglia morpho-functionality in Parkinson’s disease (PD), cerebellar function appears to be unaffected by the disease. Only recently this brain structure has been proposed to play compensatory roles in PD-induced motor dysfunction, particularly during the initial asymptomatic stages of PD. In PD subjects and animal models of PD, such as MPTP-treated mice, brain structures other than basal ganglia are also affected by the disease, including cortical areas not involved in motor control. Thus, it is noteworthy that the cerebellum remains unaffected. In the present study, we have analyzed the lipid composition of membrane microdomains [lipid rafts (LR) and non-raft domains] and assessed the expression levels of genes encoding enzymes synthesizing membrane-related lipids. The outcomes revealed that membrane domain lipids in cerebellum are highly preserved both in control and MPTP-treated mice as compared to control animals. Likewise, only small, mostly not significant, changes were observed in the expression of lipid-related genes in the cerebellum. Indeed, most changes were related to aging rather than to the exposure to the neurotoxin. Conversely, in the same animals, lipid composition, and gene expression were dramatically altered in the occipital cortex (OC), a brain area unrelated to the control of motor function. PCR and immunohistochemical analyses of both brain areas revealed that dopamine transporter (DAT) mRNA and protein were expressed in OC but not in the cerebellum. As MPTP neurotoxicity requires the expression of DAT to access intracellular compartments, we hypothesized that the absence of DAT in cerebellum hampers MPTP-induced toxicity. We conclude that cerebellum is endowed with efficient mechanisms to preserve nerve cell lipid homeostasis, which greatly maintain the stability of membrane microdomains involved in synaptic transmission, signal transduction, and intercellular communication, which together may participate in the compensatory role of the cerebellum in PD symptomatology.

## Introduction

The etiology of idiopathic Parkinson’s disease (PD) remains unknown. It is known that idiopathic PD is more prevalent in aging populations (Van Den Eeden et al., 2003; Rodriguez et al., 2015), but little is known on the involvement of other potential factors as well as on the onset of the disease. Intense research on the last decades indicates that genetic/epigenetic susceptibility along with environmental and nutritional factors are all contributors to the development of PD (Kidd, 2000; Jadiya and Nazir, 2012; Dardiotis et al., 2013; Deng et al., 2018). Compelling evidence indicates that disruption of complex 1 of electron transport chain involved in mitochondrial respiration in dopaminergic neurons represents a contributing factor to idiopathic PD (Parker et al., 1989; Swerdlow et al., 1996; Perier et al., 2007). One of the best accepted animal models for sporadic PD is the MPTP (1-methyl-4-phenyl-1,2,3,6-tetrahydropyridine) paradigm in mice, which develop signs, and symptoms of idiopathic PD (reviewed in Marsden and Sandler, 1986; Langston, 2017). These include a massive destruction of neurons at the substantia nigra pars compacta (SNpc) in the nigrostriatal pathway (Ricaurte et al., 1986). The mechanism of toxicity is based on the fact that this neurotoxin is a substrate for the dopamine transporter (DAT). Once in the nerve cell its conversion to MPP^+^ (1-methyl 4-phenylpyridinium), it provokes a specific inhibition of mitochondrial complex 1 and subsequent oxidative stress, which eventually leads to cell death (Shoffner et al., 1991; Gerlach and Riederer, 1996; Meredith and Rademacher, 2011).

One main target of such uncontrolled oxidative stress are membrane lipids, whose disruption by lipoperoxidation is tightly linked to anomalies in nerve cell physiology, including membrane fluidity and permeability, microdomain dynamics, membrane protein-protein and lipid-protein interactions, intercellular communication, signal transduction, synaptic transmission, and also to neurodegeneration (Halliwell, 1992; Götz et al., 1994). Recent studies have demonstrated that alterations in the lipid composition of membrane microdomains, particularly in lipid rafts (LR), occur in PD, even at early stages of the disease (Fabelo et al., 2011; Marin et al., 2016, 2017). Further, altering lipid composition of LR impacts the dynamics of signalosomes residing in these nanostructures and leads to aberrant signal transduction involved in neuroprotection, and promotes the redistribution of membrane proteins involved in neurotoxic cascades between rafts and non-raft domains (Fabelo et al., 2014; Marin and Díaz, 2014; Canerina-Amaro et al., 2017).

Though PD is mainly linked to degeneration of brain structures involved in motor control, i.e., basal ganglia, more recent evidence from human patients and animal models, indicate that other brain structures are also selectively affected during the progression of the disease. Indeed, cognitive impairment occurs in the majority of cases with advanced PD, and more important, altered cortical function has been frequently detected before the appearance of motor symptoms (Metzler-Baddeley, 2007; Wallin et al., 2007; Tessa et al., 2008; Ferrer, 2009). In this order of evidences, a recent review by Giguère et al. (2018) reveals that vulnerability of neuronal populations in PD extends well beyond basal ganglia and SNpc, these including the hippotalamus, locus coeruleous, pre-supplementary and premotor cortex, amygdala, thalamus, ventral tegmental area, raphe nuclei, dorsal motor nucleus of the vagus nerve, amongst others. Interestingly, no cell loss has been observed in the cerebellum. Indeed, the cerebellum and basal ganglia are the two major subcortical structures that influence motor control (Strick et al., 2009). The cerebellum is important in programing and executing movements, and is also involved in generating accurate timing of movements needed for self-paced movements. Basal ganglia and cerebellar were long assumed to be entirely separate anatomical structures and to perform distinct functional operations. However, it is known now that both structures form multi-synaptic loops with the cerebral cortex and have connections with overlapping cortical areas (Middleton and Strick, 2001; Wu and Hallett, 2013). Further, the cerebellum is known to influence motor and cognitive operations through the cerebello-thalamo-cortical circuit (Middleton and Strick, 2001). It is likely that the increased activity or connectivity in the cerebello-thalamo-cortical loop can compensate for hypofunction in the striato-thalamo-cortical circuit to maintain motor function at a near normal level. Some findings support the compensatory role of the cerebellum in Parkinson’s disease (reviewed in Wu and Hallett, 2013). We expect that such compensatory role of cerebellum lays on the stability of neuronal function in this structure and that synaptic connectivity and signaling remain unaffected during the progression of PD.

In the present study we have aimed at analyzing the potential changes on lipid composition of membrane microdomains and lipid-related gene expression in cerebellar preparations from control and MPTP mice during aging. As aging is a main risk factor for PD development, we set it as a main factor on the effects of the neurotoxin and analyzed their putative interaction. For comparative purposes, we also assessed the effects of these two factors in the occipital cortex (OC) from the same animals.

## Materials and Methods

### Animal Treatment

C57BL/6 mice were bred and maintained under standard housing conditions in a 12-h dark–light cycle at the Animal Facilities Service from University of La Laguna (Spain). Mice were fed with standard diet (AAIN93) and had free access to water. Animals were allowed to grow for 6 or 14 months. Twenty days before sacrifice animals were administered an interscapulum injection of either MPTP (MPTP-treated group) or PBS (Control group). MPTP (Sigma-Aldrich, Germany) was dissolved in PBS (0.9%) just before use and injected subcutaneously (20 mg/kg/day) once a day on 10 consecutive days, which is considered a subchronic MPTP regime (Meredith and Rademacher, 2011). After another 10 days without any manipulations, animals were sacrificed by cervical dislocation. Brain areas under study were isolated from both hemispheres. Right hemisphere was used for gene expression analyses and the left hemisphere for the isolation of rafts and non-raft fractions. In the case of immunohistochemical analyses, whole brains were processed as indicated below. All procedures were performed and authorized by the Ethics Committee at the Universidad de La Laguna. A maximum of eight animals were used per group.

### Isolation of Lipid Rafts and Non-raft Fractions

Lipid raft and non-raft fractions were isolated following the protocol described in Fabelo et al. (2012, 2014). Briefly, 0.1 g of cerebellar or occipital cortices were homogenized at 4°C in 8 volumes of isolation buffer (IB: 50 mM *Tris-HCl*, pH 8.0, 10 mM MgCl_2_, 0.15 M NaCl) containing 1% Triton X-100 and 5% glycerol, 20 mM NaF, 1 mM Na_3_VO_4_, 5 mM β-mercaptoethanol, 1 mM PMSF, and a cocktail of protease inhibitors (Roche Diagnostics, Barcelona, Spain) in a glass homogenizer for 5 min, centrifuged at 500 g for another 5 min, and then the supernatant was collected and mixed in an orbital rotor for 1 h at 4°C. About 800 μL of the supernatant was mixed with an equal volume of 80% sucrose in IB and overlaid with 7.5 mL of a 36% sucrose solution and 2.7 mL of a 15% sucrose solution in IB, in 10 mL ultracentrifuge tubes (Ultraclear, Beckman). Sucrose gradients were centrifuged at 150,000*g* for 18h at 4°C using a Beckman SW41Ti rotor. Two mL fractions were collected from the top to the bottom. Fractions 1 and 2, which contained the lipid raft resident protein markers, were pooled together and identified as lipid raft fractions. Non-raft fractions corresponded to fraction 6 and the pellet which contained the non-raft resident protein markers. Fractions were frozen at -80°C until analysis.

### Lipid Analyses

Lipid analyses were performed as described previously (Fabelo et al., 2012). Briefly, total lipids from membrane fractions were extracted with chloroform/methanol (2:1 v/v) containing 0.01% of butylated hydroxytoluene as antioxidant. Lipid classes were separated by one-dimensional double development high performance thin layer chromatography (TLC) using methyl acetate/isopropanol/chloroform/methanol/0.25% KCl (5:5:5:2:1.8 volume basis) as the developing solvent system for the polar lipid classes, and hexane/diethyl ether/acetic acid (22.5:2.5:0.25 volume basis) as the developing solvent system for the neutral lipid classes. Lipid classes were quantified by scanning densitometry after charring plates with 3% (w/v) aqueous cupric acetate containing 8% (v/v) phosphoric acid, using a Shimadzu CS-9001PC dual wavelength spot scanner.

Fatty acids composition was determined from total lipids in the fractions upon acid-catalyzed transmethylation for 16 h at 50°C, using 1 ml of toluene and 2 mL of 1% sulfuric acid (v/v) in methanol. The resultant fatty acid methyl esters (FAME) and dimethyl acetals (DMA) which originate from the 1-alkenyl chain of plasmalogens, were purified in thin layer chromatography (TLC), and quantified using a TRACE GC Ultra (Thermo Fisher Scientific, Waltham, MA, United States) gas chromatograph equipped with a flame ionization detector. Individual FAME and DMA were identified by reference to a multi-standard mixture (Supelco PARK, Supelko, Bellefonte, United States), and confirmed using a DSQ II mass spectrometer (Thermo Fisher Scientific, Waltham, MA, United States).

### Gangliosides Detection by Slot Blot Analyses

Analysis of gangliosides distribution in LR and non-rafts (NR) was performed by slot blot. Corresponding volumes for 200 ng of total protein for each experimental group were transferred to a PVDF membrane using a Slot-blot set-up (Bio-Rad). Membranes were blocked with TBS containing 3.5% (w/v) of bovine serum albumin (BSA). GM1, GD1a, GD1b, and GT1b were detected in independent membranes. The immunodetection of GD1a, GD1b, and GT1b gangliosides was performed by incubation with specific mouse monoclonal antibodies overnight at 4°C, followed by incubation with the corresponding secondary-HRP antibody. The detection of GM1 was performed by incubation with cholera toxin B subunit-HRP (1/20000, Sigma Aldrich) for 45 min at RT. Signal was developed with Clarity^TM^ Western ECL Substrate and detection was performed with Chemie-Doc MP Imaging System (Bio-Rad).

### Western Blot Analyses

For further analysis of lipid raft purity, we performed immunoblotting using different antibodies directed against lipid raft and non-raft proteins. First, equal amount of protein extracts were electrophoresed in SDS-PAGE gels, and transferred to polyvinyl (PVDF) membranes using the *Trans*-Blot Turbo rapid western blotting transfer system (Bio-Rad, Madrid, Spain). The membranes were incubated overnight at 4°C with the different primary antibodies used in this study: Mouse monoclonal anti-NeuN neuronal biomarker antibody diluted 1: 2,000; mouse monoclonal anti-PrP antibody diluted 1: 500; mouse monoclonal anti-GAPDH antibody diluted 1: 5,000; and rabbit polyclonal anti-flotillin 1 antibody diluted 1: 1,000). All antibodies were diluted in BLOTTO (5% non-fat dried milk in TBS). Following antibody incubation, membranes were washed three times for 5 min in TBS with 0.1% Tween-20. Reaction with peroxidase-conjugated anti-mouse or anti-rabbit secondary antibody was performed diluting the antibodies 1: 5,000 in BLOTTO for 1 h at room temperature. Specific bands were developed with Clarity^TM^ Western ECL Substrate and processed using Chemie-Doc MP Imaging System (Bio-Rad). The optical density was analyzed using Image Lab software.

### Relative Quantification of Gene Expression by Real-Time RT-PCR

Relative mRNA levels were calculated from Cq data following an efficiency-correction model as described previously (Díaz et al., 2016). A total of 12 mouse genes related with lipid biosynthesis were analyzed, these including genes encoding for Acetyl-Coenzyme A acetyltransferases (Acat1, Acat2, and Acat3), 3-hydroxy-3-methylglutaryl-Coenzyme A reductase (HMG-CoAR), stearoyl-Coenzyme A desaturases (Scd1 and Scd2), acyl-Coenzyme A oxidase 1 (Acox1), elongases of very long chain fatty acids (Elovl2, Elovl4, and Elovl5), and fatty acid desaturases (Fads1 and Fads2). In addition, the gene encoding the phospholipid hydroperoxide glutathione peroxidase (Gpx4 or PHGPx) was examined, specifically the mRNA species that determine the most abundant isoforms (cytosolic and mitochondrial: GPx4m+c). This antioxidant activity is essential to protect cell membrane phospholipids from oxidative damage (Casañas-Sánchez et al., 2015). The mRNA levels of two housekeeping genes (Hprt1, Polr2f, or Tbp) were used to calculate normalization factors of samples as the geometric mean of their expression values (Vandesompele et al., 2002). Optimal internal references for each comparison were selected as described by Hellemans et al. (2007). All amplification primers used in this work have been previously published (Casañas-Sánchez et al., 2015; Díaz et al., 2016).

### Immunohistochemistry

Cerebellar and occipital cortical samples were included in paraffin and 10 μm coronal sections were obtained and mounted on glass slides pre-treated with (3-Aminopropyl)triethoxysilane. Slides were deparaffinized by immersion in xylene (2× 15 min) and rehydrated using graded ethanol (2× 100% for 5 min; 95% for 5 min; 90% for 5 min; 80% for 5 min; 70% for 5 min) followed by washes with distilled water (2× 5 min) and PBS (2× 5 min). For antigen retrieval slides were boiled for 15 min in 20 mM citrate buffer (pH 6). In order to block the endogenous peroxidase activity slides were incubated for 30 min in a 0.3% H_2_O_2_ solution in PBS. After 3 washes with PBS slides were incubated overnight at RT with primary antibodies. Anti-Tyrosine Hydroxylase (Millipore, 1:500) and anti-DAT (Santa Cruz Biotechnology, 1:100). Slides were washed 3 times in PBS and then incubated 2 h at RT with the specific biotinylated secondary antibody diluted 1:200 in PBS followed by incubation with streptavidin and biotin-HRP. After washing 3 times with PBS, Signal was developed using SIGMAFAST DAB with Metal Enhancer. After washing in distilled water, slides were dehydrated in graded ethanol and xylene and permanently mounted with Eukitt (O. Kindler, Freiburg, Germany).

### Statistics

Lipid species and gene expression variables were initially assessed by one-way analyses of variance (ANOVA-I) followed by Tukey’s or Games-Howell *post hoc* tests, where appropriate. Kruskal-Wallis non-parametric test was used in cases where normality was not achieved. Comparisons between controls and MPTP-treated animals at each age were performed by using Student *t*-test or Mann-Whitney U test where appropriate. Lipid classes and main fatty acids were additionally submitted to multivariate analyses by means of principal components analysis (PCA), in order to obtain the extraction coefficient matrixes of lipid components and their contributions to overall variance and weights in group segregation. Factor scores from principal component 1 (PC1) were used to obtain the lipid profile of each experimental group. Factor scores were further analyzed by two-way ANOVA to evaluate the main effects of aging and treatment, as well as their interaction, in the lipid signatures of the different groups. For the statistical significance, a minimum of 4 animals was used for each experimental group.

## Results

### Lipid Profiles of Cerebellar Membrane Microdomains in Control and MPTP-Treated Mice

**FIGURE 1:**
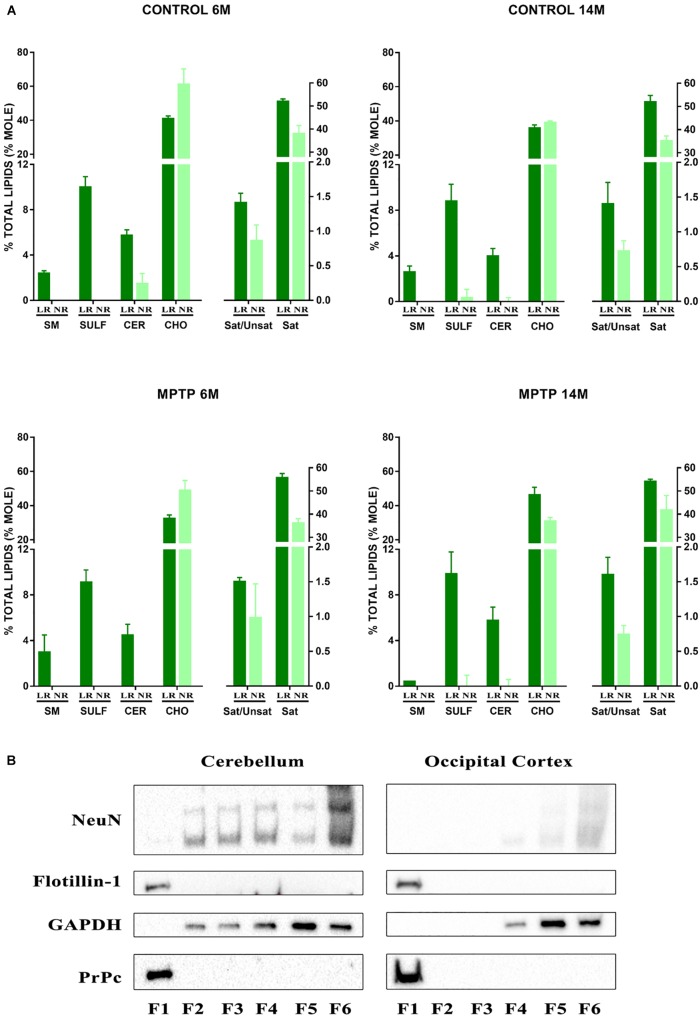
**(A)** Lipid analysis of lipid raft (LR) and non-raft (NR) fractions from cerebellum of control and MPTP-treated mice of 6 and 14 months old. Graphs show the most characteristics lipid classes and fatty acids ratios represented in LR. Remarkably sphingomyelins (SM), sulfatides (SULF), and cerebrosides (CER) are only present in LR fractions thus indicating the purity of these microdomains. CHO, cholesterol; Sat/Unsat, ratio of saturated/unsaturated fatty acids; Sat, saturated fatty acids. **(B)** Western blot analysis of fractions obtained after LR fractioning from cerebellum and occipital cortex. F1 correspond to LR microdomains. F6 corresponds to NR pellets. Both raft integrated proteins flotillin-1 and prion protein (PrPc) appear exclusively in LR microdomains thus indicating the high purity of isolation. NeuN, neuronal biomarker; GAPDH, glyceraldehyde 3-phosphate dehydrogenase.

We performed an exhaustive lipid analysis of both lipid raft and non-raft fractions isolated from cerebellum of the four experimental groups: 6-month old control, 14-month old control, 6-month old treated with MPTP, and 14-month old treated with MPTP. The purity of lipid raft extractions was demonstrated by the presence of some lipid species, such as sphingomyelin (SM), sulfatides (SULF), and cerebrosides (CER) which were exclusively present in lipid raft domains (Figure 1A, LR). We also observed differences in cholesterol (CHO) content in aged mice. Moreover, the content of proportion of saturated fatty acids (SAT) was higher in LR, also observing a higher proportion of saturated vs. unsaturated fatty acids (Sat/Unsat).

To further determine the purity of LR free of non-raft material, we performed immunoblotting assays in sample fractionation of the different total protein content. Lipid raft isolation resulted in 6 fractions (F1–F6) being F6 the insoluble pellet. Figure 1B illustrates the results obtained with control young group. Flotillin 1, the prototypical raft marker protein was exclusively present in LR (F1). Similarly, the prion protein PrP known to be integrated in neuronal LR was concentrated in F1 while the cytosolic glyceraldehyde 3-phosphate dehydrogenase (GAPDH) and neuronuclear antigen NeuN were abundant in non-raft fractions (F4–F6).

We next performed detailed analyses of lipid classes in membrane LR and NR from cerebellar samples of the different mice cohorts. Distinct lipid classes and fatty acid composition are shown in Tables 1, 2. LR contained higher levels of SAT (respectively, palmitic a (16:0) and stearic (18:0) acids) and lower contents of unsaturated (mono- and polyunsaturated) fatty acids than non-raft fractions. Within polyun SAT, arachidonic (AA, 20:4n-6), and docosahexaenoic (DHA, 22:6n-3) acids were significantly less abundant in LR. Likewise, levels of monounsaturated dimethylacetals (18:1n-7 DMA and 18:1n-7 DMA) were lower in LR. Consequently, the ratio of saturated vs. unsaturated fatty acids and the peroxidability and unsaturation indexes were lower in LR than in non-raft fractions. Conversely, non-raft fractions from control animals contained lower levels of phosphatidylethanolamine (PE) and higher contents of phosphatidylserine (PS) and phosphatidylinositol (PI). As expected, triglycerides (TG) were totally excluded in all fractions. Notably, CHO levels did not differ between fractions in either control group.

**Table 1:** Lipid class composition of lipid rafts and non-raft domains in the cerebellum of control and MPTP-treated young (6 months) and old (14 months) mice.

	Ctrl 6 months LR			Ctrl 6 months NR				Ctrl 14 months LR		Ctrl 14 months NR		

	Mean ± SEM	6 vs. 14	Ctrl vs. MPTP	Mean ± SEM	LR vs. NR	6 vs. 14	Ctrl vs. MPTP	Mean ± SEM	Ctrl vs. MPTP	Mean ± SEM	LR vs. NR	Ctrl vs. MPTP
SM	2.37 ± 0.24			0.00 ± 0.00	^∗∗∗^			2.54 ± 0.54	^∗∗^	0.00 ± 0.00		
PC	7.87 ± 0.87	^∗^	^∗^	8.53 ± 2.50				12.18 ± 0.79		11.80 ± 0.39		^∗^
PS	4.06 ± 0.61	^∗^	^∗^	6.08 ± 1.88				6.70 ± 0.45		11.53 ± 0.23	0.059	
PI	2.14 ± 0.08	^∗^		2.94 ± 0.81				2.85 ± 0.16		4.16 ± 0.08		
PG	3.15 ± 0.09			3.52 ± 0.14				2.38 ± 0.87	0.071	4.64 ± 0.43		
PE	23.76 ± 0.55			16.37 ± 4.08	^∗∗^	^∗^	0.098	24.67 ± 0.54	^∗^	27.02 ± 0.27	0.057	
SULF	9.97 ± 0.95			0.00 ± 0.00	^∗∗∗^			8.79 ± 1.49		0.31 ± 0.75		
CER	5.72 ± 0.50			1.46 ± 0.92	^∗∗∗^			3.97 ± 0.68		0.00 ± 0.34		
CHO	40.97 ± 1.59		^∗^	61.11 ± 9.12		0.075		35.76 ± 1.95	0.074	39.03 ± 0.98		
FFA	0.00 ± 0.00			0.00 ± 0.00				0.00 ± 0.00		0.52 ± 0.00	^∗∗∗^	^∗∗^
SE	0.00 ± 0.00			0.00 ± 0.00				0.16 ± 0.16		1.31 ± 0.08	^∗∗^	^∗∗∗^
TPL	59.03 ± 1.59		0.075	38.89 ± 9.12		0.081		64.08 ± 2.07	^∗^	59.45 ± 1.03		0.054

	**MPTP 6 months LR**			**MPTP 6 months NR**				**MPTP 14 months LR**		**MPTP 14 months NR**		

	**Mean** ±**SEM**	**6 vs. 14**		**Mean** ±**SEM**	**LR vs. NR**	**6 vs. 14**		**Mean** ±**SEM**		**Mean** ±**SEM**	**LR vs. NR**	

SM	2.96 ± 1.53			0.00 ± 0.00	^∗∗∗^	^∗^		0.40 ± 0.29		0.00 ± 0.14	^∗^	
PC	13.32 ± 0.77			9.49 ± 1.77	0.053	^∗^		9.79 ± 2.54		19.13 ± 1.27	^∗^	
PS	7.10 ± 0.70	0.070		8.07 ± 1.77		0.084		5.77 ± 0.82		13.44 ± 0.41	^∗^	
PI	2.99 ± 1.22			3.06 ± 0.75				3.62 ± 0.68		5.86 ± 0.34		
PG	3.01 ± 1.78			2.03 ± 1.37				0.00 ± 0.00		2.22 ± 0.00		
PE	23.97 ± 0.59	^∗^		26.98 ± 2.89				18.69 ± 0.50		26.05 ± 0.25	0.064	
SULF	9.09 ± 1.11			0.00 ± 0.00	^∗∗∗^			9.84 ± 1.93		0.00 ± 0.96		
CER	4.46 ± 0.96			0.00 ± 0.00	^∗∗∗^			5.75 ± 1.18		0.00 ± 0.59		
CHO	32.43 ± 2.16	^∗∗^		48.90 ± 5.84	0.060	^∗^		46.31 ± 4.48		30.89 ± 2.24		
FFA	0.00 ± 0.00			0.64 ± 0.64				0.29 ± 0.29		1.90 ± 0.15	^∗∗^	
SE	0.38 ± 0.38			0.85 ± 0.85				0.00 ± 0.00		0.00 ± 0.00		
TPL	66.91 ± 2.67	^∗^		49.62 ± 4.73	^∗^	^∗^		50.79 ± 2.37		66.70 ± 2.37		

*Statistical significance: ^∗^p ≤ 0.05; ^∗∗^p ≤ 0.01; ^∗∗∗^p ≤ 0.001. SM, sphingomyelin; PC, phosphatidylcholine; PS, phosphatidylserine; PI, phosphatidylinositol; PG, phosphatidylglycerol; PE, phosphatidylethanolamine; SULF, sulfatides; CER, cerebrosides; CHO, cholesterol; FFA, free fatty acids; SE, sterol esters; TPL, total polar lipids.*

**Table 2:** Main fatty acid composition of lipid rafts and non-raft domains in the cerebellum of control and MPTP-treated young (6 months) and old (14 months) mice.

	Ctrl 6 months LR			Ctrl 6 months NR				Ctrl 14 months LR		Ctrl 14 months NR		

	Mean ± SEM	6 vs. 14	Ctrl vs. MPTP	Mean ± SEM	LR vs. NR	6 vs. 14	Ctrl vs. MPTP	Mean ± SEM	Ctrl vs. MPTP	Mean ± SEM	LR vs. NR	Ctrl vs. MPTP
C 14: 0	0.70 ± 0.46			1.36 ± 1.03				0.28 ± 0.10	^∗∗∗^	0.40 ± 0.07		
C 14: 1n-5	0.17 ± 0.15			0.60 ± 0.49				0.09 ± 0.08		0.20 ± 0.06		
C 15: 0	0.37 ± 0.14			0.82 ± 0.63				0.27 ± 0.15		0.31 ± 0.08		
C 16: 0 DMA	1.07 ± 0.15			0.66 ± 0.62				1.04 ± 0.05	^∗^	1.19 ± 0.15		
C 16: 0	30.13 ± 1.95			18.77 ± 5.53	^∗^			29.49 ± 4.26		14.94 ± 2.54	^∗∗^	
C 16: 1n-9	0.71 ± 0.53			1.42 ± 0.96				0.31 ± 0.05		0.34 ± 0.05		
C 16: 1n-7	0.77 ± 0.38			1.19 ± 0.62				0.48 ± 0.02	^∗^	0.73 ± 0.18		
C 16: 2n-4	0.05 ± 0.08			0.39 ± 0.36				0.00 ± 0.00		0.00 ± 0.00		
C 17: 0	0.27 ± 0.07			0.49 ± 0.29				0.23 ± 0.04		0.24 ± 0.03		
C 18: 0 DMA	3.30 ± 0.19			2.39 ± 1.40				3.25 ± 0.08	^∗^	3.30 ± 0.20		
C 18: 1n-9 DMA	0.48 ± 0.05			0.67 ± 0.66				0.56 ± 0.25		1.28 ± 0.22		
C 18: 1n-7 DMA	0.72 ± 0.21			1.63 ± 0.17	0.06			0.91 ± 0.23		1.80 ± 0.19	^∗^	
C 18: 0	21.82 ± 0.34			19.21 ± 1.63	^∗^			22.39 ± 1.46		20.13 ± 1.90		
C 18: 1n-9	13.95 ± 0.69			14.00 ± 1.83				13.02 ± 1.02		14.38 ± 0.46		
C 18: 1n-7	5.40 ± 0.56			3.85 ± 1.17				5.46 ± 0.32		4.45 ± 0.17		
C 18: 1n-5	0.81 ± 0.22			3.23 ± 1.90				0.73 ± 0.19		1.79 ± 0.08	^∗^	
C 18: 2n-6	0.82 ± 0.23			1.98 ± 0.41	^∗^			0.60 ± 0.10		1.12 ± 0.03	^∗^	
C 18: 3n-6	0.21 ± 0.21			0.45 ± 0.43				0.09 ± 0.08		0.14 ± 0.12		
C 18: 3n-3	0.00 ± 0.00			0.00 ± 0.00				0.00 ± 0.00		0.09 ± 0.16		
C 20: 0	0.31 ± 0.06			0.51 ± 0.35				0.24 ± 0.07		0.32 ± 0.10		
C 20: 1n-9	1.54 ± 0.28			1.02 ± 0.21	^∗^			1.23 ± 0.07		0.99 ± 0.01	^∗^	
C 20: 1n-7	0.39 ± 0.04			0.05 ± 0.09				0.34 ± 0.02		0.26 ± 0.01	^∗^	
C 20: 3n-6	0.22 ± 0.03			0.41 ± 0.40				0.18 ± 0.07		0.64 ± 0.03		
C 20: 4n-6	4.04 ± 0.65			5.75 ± 3.70				4.80 ± 1.39		8.58 ± 0.97	^∗∗^	
C 20: 5n-3	0.05 ± 0.09			1.18 ± 2.04				0.00 ± 0.00		0.06 ± 0.10		
C 22: 0	0.12 ± 0.12			0.00 ± 0.00				0.09 ± 0.08		0.00 ± 0.00		^∗^
C 22: 4n-6	0.64 ± 0.10			0.76 ± 0.72				0.69 ± 0.22		1.48 ± 0.25	^∗∗^	
C 22: 5n-6	0.04 ± 0.06			0.06 ± 0.10				0.00 ± 0.00		0,00 ± 0.00		
C 22: 6n-3	8.99 ± 2.16			12.26 ± 6.76	0.05			11.40 ± 2.32		18.28 ± 2.81	^∗∗^	
C 24: 1n-9	0.69 ± 0.19			0.49 ± 0.62				0.57 ± 0.09		0.31 ± 0.09	^∗^	
Totals and indexes												
SFAs	58.22 ± 2.56			44.21 ± 5.96	^∗^			57.36 ± 5.91		40.93 ± 4.49	^∗∗^	
UFAs	41.37 ± 2.10			52.64 ± 8.09				41.82 ± 5.67		57.69 ± 5.07	^∗∗^	
MUFAs	26.26 ± 1.45			28.96 ± 1.53			^∗^	23.88 ± 1.57		26.92 ± 1.03	^∗^	
PUFAs	15.12 ± 2.55			23.68 ± 9.61				17.94 ± 4.12		30.77 ± 4.23	^∗∗^	
Sat/Unsat	1.41 ± 0.14			0.86 ± 0.23	^∗∗^			1.40 ± 0.31		0.72 ± 0.15	^∗^	
DMAs	5.57 ± 0.55			5.35 ± 2.57				5.77 ± 0.49	^∗^	7.56 ± 0.72	^∗^	
PI	88.92 ± 18.77			132.22 ± 60.83				109.82 ± 23.94		181.96 ± 27.86	^∗∗^	
Ul	102.50 ± 14.14			144.11 ± 49.98				116.75 ± 22.48		183.51 ± 23.81	^∗∗^	
C 14: 0	0.41 ± 0.08			0.45 ± 0.31				0.49 ± 0.10	^∗∗∗^	0.31 ± 0.09		
C 14: 1n-5	0.13 ± 0.14			0.30 ± 0.13				0.30 ± 0.09		0.22 ± 0.12		
C 15: 0	0.36 ± 0.11			0.35 ± 0.28				0.41 ± 0.08		0.29 ± 0.04	^∗^	
C 16: 0 DMA	0.96 ± 0.08			1.09 ± 0.13				0.87 ± 0.02	^∗^	1.12 ± 0.24		
C 16:0	32.42 ± 2.30			15.16 ± 2.50	^∗∗^			31.86 ± 1.51		20.44 ± 11.21	^∗∗^	
C 16: 1n-9	0.34 ± 0.04			0.38 ± 0.17				0.40 ± 0.06		0.37 ± 0.09		
C 16: 1n-7	0.68 ± 0.23			0.88 ± 0.25				0.65 ± 0.05	^∗^	0.68 ± 0.02		
C 16: 2n-4	0.02 ± 0.04			0.00 ± 0.00				0.00 ± 0.00		0.00 ± 0.00		
C 17: 0	0.28 ± 0.04			0.24 ± 0.09				0.27 ± 0.02		0.19 ± 0.05		
C 18: 0 DMA	3.01 ± 0.13			3.36 ± 0.34				2.76 ± 0.12	^∗^	3.22 ± 0.61		
C 18: 1n-9 DMA	0.38 ± 0.15			1.12 ± 0.15	^∗∗^			0.44 ± 0.04		0.54 ± 0.60		
C 18: 1n-7 DMA	0.71 ± 0.17			1.53 ± 0.10	^∗^			0.74 ± 0.06		1.31 ± 0.54	^∗^	
C 18: 0	23.14 ± 1.65			20.91 ± 1.52				22.17 ± 0.58		21.27 ± 1.55		
C 18: 1n-9	12.57 ± 1.04			14.02 ± 1.75				11.96 ± 0.49		13.86 ± 2.19		
C 18: 1n-7	5.39 ± 0.37			4.43 ± 0.56	0.07			5.36 ± 0.18		4.95 ± 0.51	^∗^	
C 18: 1n-5	0.90 ± 0.60			2.89 ± 1.73				1.17 ± 0.25		1.54 ± 0.68		
C 18: 2n-6	0.84 ± 0.33			1.22 ± 0.23				0.83 ± 0.20		1.18 ± 0.16	^∗^	
C 18: 3n-6	0.08 ± 0.08			0.16 ± 0.27				0.22 ± 0.06		0.24 ± 0.17		
C 18: 3n-3	0.06 ± 0.10			0.13 ± 0.23				0.06 ± 0.10		0.00 ± 0.00		
C 20: 0	0.27 ± 0.04			0.24 ± 0.13				0.27 ± 0.08		0.23 ± 0.11		
C 20: 1n-9	1.38 ± 0.27			0.87 ± 0.12	^∗^			1.22 ± 0.12		1.01 ± 0.20	^∗^	
C 20: 1n-7	0.38 ± 0.06			0.27 ± 0.08				0.33 ± 0.03		0.27 ± 0.05	^∗^	
C 20: 3n-6	0.11 ± 0.09			0.54 ± 0.21	^∗^			0.13 ± 0.22		0.57 ± 0.24	0.07	
C 20: 4n-6	3.79 ± 0.21			8.34 ± 1.51	^∗∗^			3.88 ± 0.29		7.35 ± 3.01	^∗∗^	
C 20: 5n-3	0.09 ± 0.16			0.00 ± 0.00				0.06 ± 0.10		0.08 ± 0.15		
C 22: 0	0.11 ± 0.10			0.00 ± 0.00				0.11 ± 0.10		0.06 ± 0.10		^∗^
C 22: 4n-6	0.55 ± 0.12			1.32 ± 0.23	^∗^			0.58 ± 0.08		1.18 ± 0.56	^∗^	
C 22: 5n-6	0.08 ± 0.08			0.14 ± 0.12				0.00 ± 0,00		0.04 ± 0.07		
C 22: 6n-3	9.43 ± 1.17			17.44 ± 2,98	^∗∗^			9.88 ± 0.59		15.25 ± 5,37	^∗∗^	
C 24: 1n-9	0.35 ± 0.32			0.18 ± 0,17				0.59 ± 0.20		0,21 ± 0.20		
Totals and indexes												
SFAs	60,96 ± 3.40			41.88 ± 4.19	^∗∗^			59.39 ± 0,87		47.15 ± 11.25	^∗∗^	
UFAs	38.52 ± 3.71			56.90 ± 4.60	^∗∗^			39.57 ± 0.98		51.81 ± 11.28	^∗∗^	
MUFAs	23.42 ± 1.56			27.46 ± 0.52	^∗∗^	^∗^	^∗^	23.65 ± 0.32		25.46 ± 1.74	^∗^	
PUFAs	15.10 ± 2.15			29.44 ± 4.37	^∗∗^			15.93 ± 0.66		26.35 ± 9.59	^∗∗^	
Sat/Unsat	1.60 ± 0.25			0.74 ± 0.13	^∗^			1.50 ± 0.06		0.98 ± 0.49	^∗^	
DMAs	5.06 ± 0.48			7.10 ± 0.70	^∗^			4.80 ± 0.21	^∗^	6.19 ± 1.33	^∗^	
PI	91.54 ± 12.12			173.46 ± 29.19	^∗∗∗^			95.29 ± 5.60		153.34 ± 56.17	^∗∗^	
Ul	100.82 ± 11.91			176.91 ± 24.36	^∗∗∗^			104.51 ± 4.64		158.06 ± 50.60	^∗^	

*Statistical significance: ^∗^p ≤ 0.05; ^∗∗^p ≤ 0.01; ^∗∗∗^p ≤ 0.001. SFAs, saturated fatty acids; UFAs, unsaturated fatty acids; MUFA**s**, monosaturated fatty acids; Sat/Unsat, ratio saturated versus unsaturated fatty acids; DMAs, dimethyl acetal fatty acids; PI, peroxidability index; UI, unsaturation index.*

**FIGURE 2:**
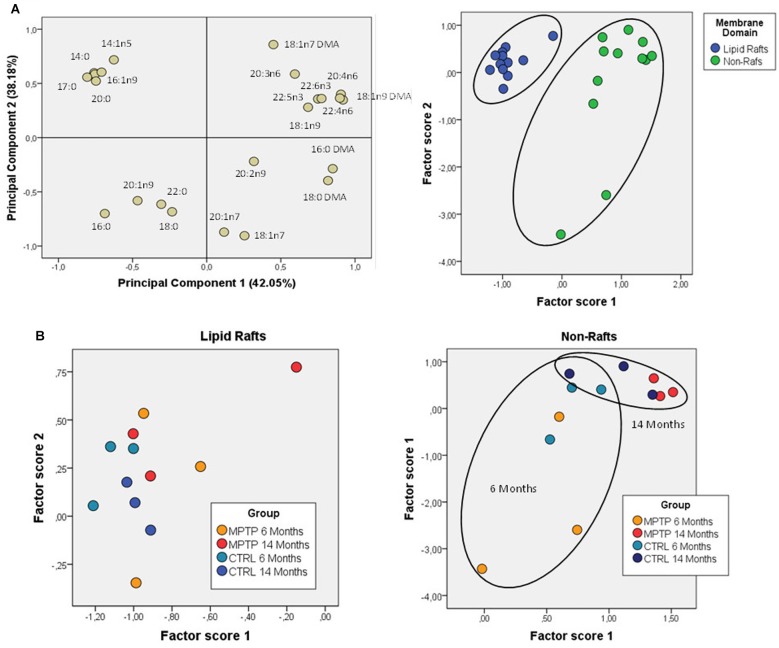
Lipid classes profile of cerebellar membrane microdomains in control and MPTP-treated mice. **(A)** Principal components analyses of lipid classes’ distribution. Left plot, loading scores for the lipid class included in the PCA. In parentheses the percent of total variance explained by each component. CHO, cholesterol; CER, cerebrosides; SULF, sulfatides; SM, sphingomyelins; PG, phosphatidylglycerol; Pi, phosphatidylinositol; PS, phosphatidylserine; PE, phosphatidylethanolamine, PC, phosphatidylcholine; SE, sterol esters; TPL, total polar lipids; FFA, free fatty acids. Right plot, factor scores grouped by membrane domain. **(B)** Plots of factor scores for the four different groups for LRs (right) and NRs (left), respectively. 4–8 animals were used for each experimental group.

**FIGURE 3:**
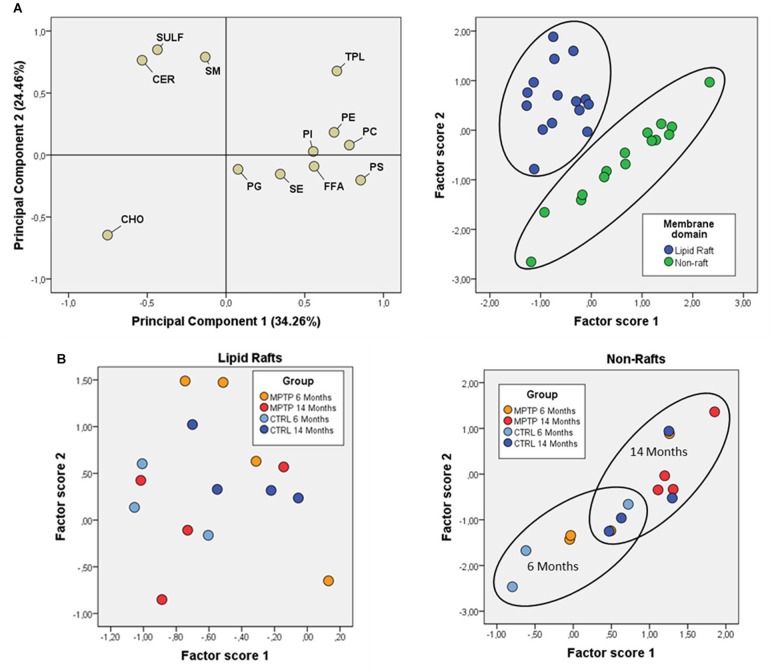
Fatty acid contents in polar lipids from cerebellar membrane microdomains in control and MPTP-treated mice. **(A)** Principal components analyses of fatty acids distribution. Left plot, loading scores for the fatty acids included in the PCA. In parentheses the percent of total variance explained by each component. Right plot: Factor scores grouped by lipid domain. **(B)** Plots of factor scores for the four different groups from LRs (right) and NRs (left), respectively. 4–8 animals were used for each experimental group.

Further comparative distribution of lipid classes in membrane microdomain partitioning was determined by multivariate analysis of the most representative lipid classes in cerebellum (Figure 2A). We used a multifactorial approach in the aim to delineate the potential interactions of both age and neurotoxic treatment factors. For comparative analyses, we also established equivalent studies in occipital cortices of the same mice cohorts (Figure 3A). Multivariate analyses revealed that the lipid fingerprints of LR and non-raft fractions in the whole set of data was sufficiently different as to allow a perfect segregation of the two domains. Based on factor analyses, this segregation was explained by two principal components, which together explained 80.23 and 58.72% of total variance for fatty acids and lipid classes, respectively. In the case of fatty acids, principal component 1 (PC1) was positively related to main polyunsaturates (AA, DHA, docosatetra- and docosapenta-enoic acids) and dimethylacetals (DMA, both saturates and monounsaturates) which were more abundant in non-raft fractions, and negatively to saturates [myristic (14:0), palmitic (16:0), magaric (17:0), arachidic (20:0) acids], more abundant in LR. Principal component 2 (PC2) was related positively to 18:1n-7 DMA (mainly) and negatively to stearic (18:0) and to 20- and 18- carbon atoms n-7 fatty acids. In the case of lipid classes, principal component 1 (PC1) was positively related to phospholipids PE, PC, PS, and PI, which were more abundant in non-raft fractions, and negatively to CHO, which differ between fractions depending on the treatment. In the case of PC2, levels of SULF, CER, and SM (nearly exclusively present in LR) were positively related, and negatively to CHO (as in the case of PC1).

Comparison of factor scores 1 and 2 for fatty acids indicated that lipid profiles in the two fractions were significantly different for factor 1 (*F* = 140.71, *p* = 0.000), but not for factor 2 (*F* = 1.32, *p* = 0.262). In the case of lipid classes, fractions were again different with both factor scores being statistically significant (Factor 1: *F* = 31.39, *p* = 0.000; Factor 2: *F* = 8.99, *p* = 0.006).

Overall, these results demonstrate that fraction 1 corresponds to purified LR.

**FIGURE 4:**
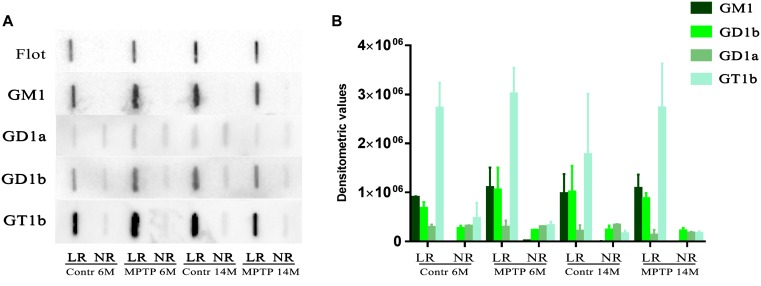
Slot-blot analysis of gangliosides in LR and non-raft (NR) fractions from cerebellum of both control and MPTP treated mice of 6 months (6M) and 14 months (14 M) old. **(A)** Slot blot to detect four main brain gangliosides using specific antibodies directed to each of the ganglioside species, GM1, GD1a, GD1b, and GT1b. For comparison and control of raft purity, flotillin-1 was also included. **(B)** Statistical quantification of slot-blotting immunosignals of the different cohorts. Notice that flotillin-1 and GM1 are only present in LR fractions as an indicator for LR purity.

Gangliosides are one of the most representative lipid classes in LR but are not properly isolated by sucrose gradients in the presence of detergent as in the present study. Therefore, we next analyzed ganglioside profiles by slot blotting experiments using lipid raft and non-raft samples to blot against specific antibodies directed to the main ganglioside species (GM1, GD1a, GD1b, and GT1b). The pattern of distribution between lipid raft and non-raft fractions of control and MPTP-treated mice indicated that GM1, GD1b, and GT1b were mainly represented in LR (Figure 4A). In contrast, GD1a was faintly shown in these fractions, not observing significant differences between microdomain partitioning in the different cohorts. Moreover, flotillin-1 as a main representative protein of LR was used for comparison, observing the presence of this marker exclusively in raft fractions. Figure 4B shows the quantification of immunosignals corresponding at each of the ganglioside species in LR as compared to NR. The results indicate that gangliosides are highly enriched in lipid raft fractions. Noticeably, both GM1 and flotillin 1 were exclusively represented in raft structures.

### Effects of Aging and MPTP-Neurotoxic Treatment on Membrane Lipid Microdomains of the Cerebellum

We next characterized the distribution pattern of lipid classes and fatty acids in the different experimental groups (aged and MPTP-treated mice). When comparing cerebellar lipid profiles of aged and neurotoxin-treated animals, it became evident that these two variables were interrelated. Therefore, in order to detect the potential differences in lipid matrices during aging and neurotoxic treatment we performed principal component analyses for both fatty acids and lipid classes (Figure 2B, 3B, respectively). The results showed that in control animals, aging was accompanied by subtle changes in cerebellar microdomains in lipid classes but not in the fatty acid composition (Figure 2B). Thus, in LR, levels of PC, PS and PI were significantly augmented in 6-months mice compared to 14 months (Figure 2B). In non-raft fractions, significant changes were only observed for PE, although levels of CHO (higher in 6-months old mice) and total polar lipids (TPL) (higher in older mice) were close to statistical significance.

The effects of MPTP-treatment in cerebellar lipid matrix were strongly dependent on mice age. Thus, at the age of 6 months, MPTP caused a significant increase in PC and PS, and a reduction of CHO in LR, while in non-raft fractions no significant changes were detected (Figure 3B). In non-raft fractions, most significant changes were observed for PC, PS, and TPL (higher in 14 months-old mice), and CHO (higher in younger animals). No changes were observed in the fatty acid composition of non-raft fractions as a function of aging. In contrast, at the age of 14 months, LR from MPTP-treated animals exhibited lower levels of SM, PG, and TPL, as well as significant increased CHO levels (*p* = 0.074). Moreover, there was a reduction in TPL and PE (and PS, *p* = 0.07) in older mice treated with the neurotoxin (Figure 3B). Regarding non-raft fractions, MPTP-treatment was associated to increased PC, FFA, and SE only in 14 month-old mice (see Table 1).

### Effects of Aging and MPTP-Neurotoxic Treatment on the Lipid Content of the Occipital Cortex

We next explored whether either age or MPTP treatment may affect the lipid content of the OC, a brain area unrelated to the control of motor function. Thus, we established the lipid profile of this brain area in the different experimental mice (Table 3). In control animals, aging was responsible for a reduction of SE and an increase in CHO levels, as well as a significant reduction in phospholipid-to-cholesterol ratio. Significant changes were also observed in some minor fatty acids as a consequence of aging (15:0, 16:1n-7 and 18:1n-7 DMA). Unlike cerebellum, MPTP-treatment brought about age-dependent lipid alterations in OC. Thus, in aged animals, the neurotoxin increased the levels of SE, 18:1n-7 DMA, 18:1n-7 DMA, 18:1n-9, 18:2n-6, 20:2n-6 and 24:1n-9, and reduced levels of FFA and 22:5n-6. Within totals and indexes, MPTP-treatment increased total MUFA and total DMA (plasmalogens), and reduced total polyunsaturated fatty acids (PUFA) and phospholipid-to-cholesterol ratio. Moreover, OC in older mice exhibited higher SULF, cholesterol, SE, 15:0, and 18:2n-6 as well as lower TPL and FFA.

**Table 3:** Lipid classes and main fatty acids composition in the occipital cortex of control and MPTP-treated young (6 months) and old (14 months) mice.

	Ctrl 6 months LR	Ctrl 6 months NR		Ctrl 14 months LR		Ctrl 14 months NR		

	Mean ± SEM	Mean ± SEM	6 vs. 14	Mean ± SEM	Ctrl vs. MPTP	Mean ± SEM	6 vs. 14	Ctrl vs. MPTP
**Lipid classes**								
SM	1.25 ± 0.08	1.47 ± 0.22		1.18 ± 0.15		0.91 ± 0.08		
PC	11.89 ± 0.37	10.68 ± 0.72		10.67 ± 0.42	0.10	10.05 ± 0.60		
PS	12.35 ± 0.65	11.93 ± 0.86		11.34 ± 0.16		10.52 ± 1.09		
PI	2.83 ± 0.14	2.64 ± 0.34		2.54 ± 0.12		2.91 ± 1.04		
PG	3.05 ± 0.29	2.81 ± 0.18		3.07 ± 0.14		2.49 ± 0.45		
PE	25.56 ± 0.40	25.83 ± 0.29		25.47 ± 0.63		24.10 ± 0.19		
SULF	7.94 ± 0.28	9.35 ± 1.21		9.12 ± 0.54		10.31 ± 0.10	0.10	^∗^
CER	3.02 ± 0.50	2.71 ± 0.44		3.32 ± 0.27		3.59 ± 0.11		
CHO	29.84 ± 0.39	31.53 ± 0.41	^∗^	32.27 ± 1.42	0.08	34.43 ± 0.63		^∗^
FFA	1.42 ± 0.26	0.69 ± 0.29		1.02 ± 0.01		0.08 ± 0.03	^∗∗^	^∗∗^
TG	0.00 ± 0.00	0.00 ± 0.00		0.00 ± 0.00		0.00 ± 0.00		
SE	0.85 ± 0.07	0.54 ± 0.05	^∗^	0.00 ± 0.00	^∗∗^	0.59 ± 0.09	^∗∗∗^	^∗^
TPL	67.89 ± 0.54	67.42 ± 0.20		66.70 ± 1.42		64.89 ± 0.53		^∗^
Phosphol/CHO	1.87 ± 0.03	1.72 ± 0.04	^∗^	1.65 ± 0.11		1.46 ± 0.04	0.05	
Fatty acids								
C 14: 0	0.20 ± 0.07	0.14 ± 0.01		0.15 ± 0.02		0.14 ± 0.00		
C 15: 0	0.09 ± 0.04	0.04 ± 0.01	^∗∗^	0.06 ± 0.01		0.06 ± 0.00		^∗^
C 16: 0 DMA	1.98 ± 0.17	1.98 ± 0.14		1.92 ± 0.12		1.79 ± 0.04		^∗^
C 16: 0	20.97 ± 0.77	20.80 ± 0.52		20.78 ± 0.29		20.38 ± 0.49		
C 16: 1n-9	0.22 ± 0.02	0.20 ± 0.02		0.18 ± 0.02		0.17 ± 0.01		
C 16: 1n-7	0.54 ± 0.02	0.58 ± 0.01	^∗∗^	0.55 ± 0.02		0.58 ± 0.01		
C 17: 0	0.15 ± 0.01	0.14 ± 0.01		0.15 ± 0.01		0.15 ± 0.01		
C 17: 1n-7	0.02 ± 0.04	0.00 ± 0.00		0.00 ± 0.00		0.02 ± 0,04		
C 18: 0 DMA	3.70 ± 0.15	3.50 ± 0.16		3.76 ± 0.10		3.63 ± 0.05	^∗^	
C 18: 1n-9 DMA	1.23 ± 0.13	1.35 ± 0.18		1.34 ± 0.07		1.50 ± 0.05	^∗∗^	
C 18: 1n-7 DMA	1.35 ± 0.21	1.88 ± 0.32	^∗^	1.59 ± 0.08		2.14 ± 0.10	^∗∗∗^	
C 18: 0	19.56 ± 0.29	19.58 ± 0.28		19.50 ± 0.06		19.48 ± 0.29		
C 18: 1n-9	14.35 ± 0.51	14.84 ± 1.12		15.00 ± 0.28		15.80 ± 0.10	^∗∗∗^	
C 18: 1n-7	3.26 ± 0.11	3.31 ± 0.26		3.40 ± 0.15		3.49 ± 0.04		
C 18: 2n-6	0.71 ± 0.17	0.55 ± 0.01		0.55 ± 0.02		0.66 ± 0.02	^∗∗∗^	^∗∗∗^
C 20: 0	0.22 ± 0.03	0.20 ± 0.03		0.23 ± 0.03		0.22 ± 0.01		
C 20: 1n-9	1.05 ± 0.22	1.06 ± 0.26		1.19 ± 0.09		1.30 ± 0.05		
C 20: 1n-7	0.24 ± 0.02	0.23 ± 0.04		0.25 ± 0.02		0.27 ± 0.01		
C 20: 2n-6	0.16 ± 0.01	0.10 ± 0.08		0.16 ± 0.01		0.18 ± 0.00	^∗∗∗^	
C 20: 3n-6	0.32 ± 0.01	0.31 ± 0.02		0.32 ± 0.01		0.32 ± 0.01		
C 20: 4n-6	10.26 ± 1.13	10.24 ± 1.07		9.87 ± 0.65		9.10 ± 0.25		
C 22: 0	0.19 ± 0.02	0.16 ± 0.04		0.18 ± 0.03		0.18 ± 0.01		
C 22: 1n-9	0.06 ± 0.05	0.03 ± 0.04		0.06 ± 0.05		0.03 ± 0.04		
C 22: 4n-6	2.55 ± 0.26	2.62 ± 0.23		2.56 ± 0.23		2.49 ± 0.10		
C 22: 5n-6	0.39 ± 0.10	0.27 ± 0.04		0.38 ± 0.04		0.23 ± 0.03	^∗∗∗^	
C 22: 6n-3	15.11 ± 0.41	14.78 ± 0.49		14.72 ± 0.38		14.51 ± 0.44		
C 24: 1n-9	0.73 ± 0.14	0.84 ± 0.17		0.82 ± 0.09		0.95 ± 0.04	^∗^	
Totals and indexes								
SFAs	47.14 ± 0.82	46.55 ± 0.63		46.79 ± 0.14		46.02 ± 0.73		
UFAs	52.67 ± 0.83	53.20 ± 0.63		52.95 ± 0.21		53.76 ± 0.76		
MUFAs	23.04 ± 1.38	24.31 ± 2.39		24.37 ± 0.69		26.25 ± 0.16	^∗∗^	
PUFAs	29.63 ± 0.91	28.89 ± 1.79		28.58 ± 0.69		27,51 ± 0.65	^∗^	
Sat/Unsat	0,90 ± 0.03	0.88 ± 0.02		0.88 ± 0.01		0,86 ± 0.03		
DMAs	8.26 ± 0.38	8.72 ± 0.53		8.61 ± 0.13		9.05 ± 0.22	^∗∗^	
PI	164.49 ± 1.27	160.45 ± 7.89		159.42 ± 2.09		154.48 ± 3.88		
Ul	170.22 ± 1.77	168.13 ± 5.80		166.80 ± 1.89	^∗∗^	163.56 ± 3.48		

*Statistical significance: ^∗^p ≤ 0.05; ^∗∗^p ≤ 0.01; ^∗∗∗^p ≤ 0.001. SM, sphingomyelin; PC, phosphatidylcholine; PS, phosphatidylserine; PI, phosphatidylinositol; PG, phosphatidylglycerol; PE, phosphatidylethanolamine; SULF, sulfatides; CER, cerebrosides; CHO, cholesterol; FFA, free fatty acids; TG, triglycerides; SE, sterol esters; TPL, total polar lipids; Phosphol/CHO, ratio phospholipids versus cholesterol; SFAs, saturated fatty acids; UFAs, unsaturated fatty acids; MUFAs, monosaturated fatty acids; Sat/Unsat, ratio saturated versus unsaturated fatty acids; DMAs, dimethyl acetal fatty acids; PI, peroxidability index; UI, unsaturation index.*

**FIGURE 5:**
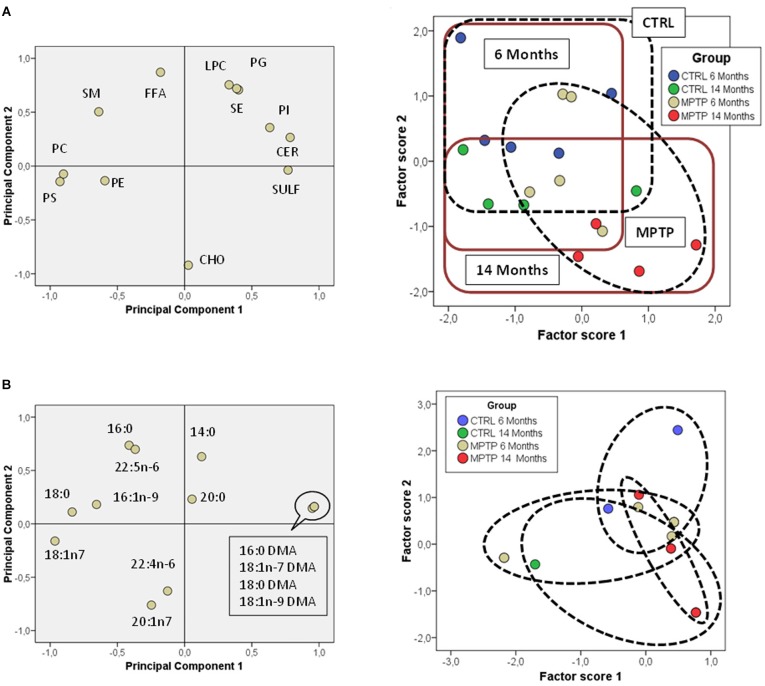
Lipid class profiles in occipital cortex from control and MPTP-treated mice. **(A)** Principal components analyses of lipid classes’ distribution. Left plot, loading scores for the lipid class included in the PCA. CHO, cholesterol; CER, cerebrosides; SULF, sulfatides; SM, sphingomyelins; PG, phosphatidylglycerol; Pi, phosphatidylinositol; PS, phosphatidylserine; PE, phosphatidylethanolamine; PC, phosphatidylcholine; SE, sterol esters; TPL, total polar lipids; FFA, free fatty acids. Right plot, factor scores grouped by lipid domain. Right plot, factor scores colored by groups. **(B)** Left plot, principal components analyses of fatty acids distribution. Loading scores for the fatty acids included in the PCA. Right plot, factor scores colored by groups. 4–8 animals were used for each experimental group.

In an attempt to further establish the potential interrelations between aging and MPTP effects on the occipital area, we performed principal component analyses on lipid classes. The results showed that the different mice groups were segregated based on their lipid composition (Figure 5A). PC1 explained 34.5% of total variance and was positively related to SULF and CER (characteristic of LR), and negatively to main phospholipids (PC and PE, more abundant in non-raft). PC2 accounted for 30.1% of total variance and was positively related to FFA and SE and negatively to CHO. Analyses of factor scores using ANOVA II revealed that main factors, age and treatment, were significantly determinant of factor score 2 (Age: *F* = 14.99 *p* = 0.002; Treatment: *F* = 6.35 *p* = 0.024) and that some degree of interaction between both factors is revealed Factor score 1 (*F* = 3.1 *p* = 0.1). Regarding fatty acids, PCA showed that the two principal components explained more than 67% of total variance, with PC1 positively related to plasmalogens (more abundant in 14-months old MPTP group) and negatively to 18:0 and 18:1n7, and PC2 positively related to saturates 16:0 and 14:0 (Figure 5B). However, no clear statistical influences of either factor score were detected for main factors alone, but their interaction was significant (*F* = 3.26; *p* = 0.048). These results indicate that the lipid composition of OC is significantly affected by the combination of both aging and neurotoxicity.

### Effects of Aging and MPTP Treatment in Lipid-Related Gene Expression of Cerebellum

**FIGURE 6:**
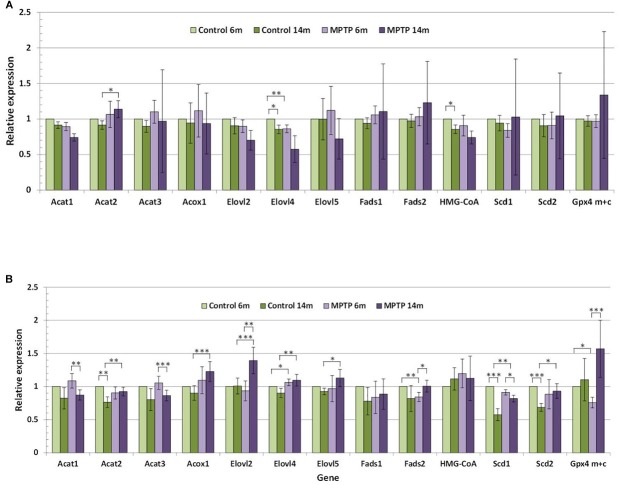
**(A)** Relative expression of lipid-related genes in cerebellum of control and MPTP-treated young (6 months) and old (14 months) mice. **(B)** Relative expression of lipid-related genes in occipital cortex of control and MPTP-treated young (6 months) and old (14 months) mice. Data correspond to the mean ± SEM. Expression level of each gene in the different samples was relativized to control young mice used as calibrator samples. Significant differences are indicated (^∗^*p* < 0.1, ^∗∗^*p* < 0.05, and ^∗∗∗^*p* < 0.01). 4 animals were used for each experimental group.

Given that CHO is one of the most abundant lipid classes in neuronal LR, we have analyzed the expression of different genes encoding CHO metabolism. These include *HMG-CoAR* (encoding for 3-hydroxy-3-methyl-glutaryl-CoA reductase, the rate-limiting enzyme in CHO biosynthesis), *Acat1-3* (encoding for Acyl-CoA CHO acyltransferase, which forms steryl/cholesteryl esters and is associated with amyloid beta production), and Scd1-2 (encoding for stearoyl-CoA desaturase, or 9 desaturase). Furthermore, we have investigated genes involved in the biosynthetic pathway for polyunsaturated fatty acids, such as *Elovl2-5* (after Elongases of very-long chain fatty acids, and encoding for elongases 2, 4 and 5), *Fads1-2* (encoding for Δ5 and Δ6 desaturases), *Acox1* (encoding for peroxisomal acyl-CoA oxidase 1, that catalyzes the chain-shortening of 24-carbon intermediates through partial β-oxidation) and *scd1* (encoding for stearoyl-CoA desaturases). Figure 6A shows the relative expression of these genes in the cerebellum of control and MPTP-treated young and older mice.

The comparative analyses of the relative expression of the different genes in the cerebellum reflected that lipid changes in mice cohorts were accompanied by alterations in the cerebellar expression levels of some lipid-related genes, in particular in aged animals (Figure 6A). It is worth mentioning that a significant reduction (*p* < 0.1) in the expression levels of *HMG-CoA* and *Elovl4* was detected in younger control animals, but not in older controls or groups of MPTP-treated mice. In control animals, some discrete changes were seen for *Acat2*, and *Scd1-2* genes, all of which were upregulated by aging. However, in MPTP-treated mice, age-related changes affected *Acat1*, *Acat3*, and *Scd1* (which were upregulated) whereas *Elovl2*, *Fads2*, and *GPx4_m+c_* were downregulated in aged animals. Related to MPTP treatment, comparison between controls and MPTP-groups showed a number of significant differences mostly in older animals. Thus, in 14-months old mice, the neurotoxin reduced the expression of *Acat2*, *Acox1*, *Elovl2*, *Elovl4* and *Scd1*, and to a lower extent *Elovl5* and *Scd2*. In contrast, only slight changes in gene expression were observed for *Acat2* (*p* = 0.087) in 14 months-old mice and *Elovl4* (*p* = 0.032) in 6-months old animals.

For comparison, we also determined the expression of lipid-related genes in the OC (Figure 6B). We found that expression levels significantly varied between these two brain areas at both ages. Indeed, we observed that cerebellum exhibits much higher expression levels of *Acat3*, *Fads2*, *HMG-CoA, Acat2, Scd2*, and *Fads1*, and lower levels of *Elovl2* and GPx4_m_*_+c_*. These trends were observed for all groups, irrespective of age and treatment.

For a better clarification of the differences in the lipid-related gene expression in both cerebellar and occipital nerve structures, we have established statistical comparison of the different experimental groups. These data are indicated in Table 4.

**Table 4:** Comparative expression of lipid-related genes between Occipital cortex (OCC) and Cerebellum (CER) in control and MPTP-treated mice at 6 and 14 months.

	Control 6 months		Control 14 months		MPTP 6 months		MPTP 14 months	
Gene	COC vs. CER		COC vs. CER		COC vs. CER		COC vs. CER	
	Expression	*p*-value	Expression	*p*-value	Expression	*p*-value	Expression	*p*-value
*Acat1*	1.057		0.949		1.199	0.004	1.215	
*Acat2*	1.481	0.032	1.234	0.006	1.176	0.057	1.178	0.004
*Acat3*	3.697	0.000	3.381	0.004	3.316	0.018	3.311	0.015
*Acoxl*	1.099		1.053		1.013		1.415	0.000
*Elovl2*	0.495	0.017	0.547	0.008	0.476	0.022	0.951	
*Elovl4*	0.894		0.948		1.028		1.595	
*Elovl5*	1.039		0.973		0.841		1.61	0.041
*Fads1*	1.726	0.000	1.439	0.002	1.28	0.077	1.361	0.013
*Fads2*	2.055	0.019	1.74	0.004	1.564	0.023	1.668	0.023
*HMG-CoA*	1.39	0.005	1.828	0.017	1.705	0.011	2.08	0.007
*Scd1*	1.03		0.625	0.027	1.041		0.8	0.037
*Scd2*	1.682	0.000	1.283	0.051	1.519	0.047	1.49	0.004
*Gpx4 m+c*	0.614	0.023	0.698	0.077	0.445	0.012	0.706	0.023

*Significant differences at p < 0.05 or p < 0.1 are highlighted in blue/red (downregulation/upregulation) or orange, respectively.*

### Effects of Aging and MPTP Treatment on the Presence of DAT and TH in Occipital Cortex and Cerebellum

It has been previously characterized that MPTP uptake by neurons occurs after oxidation of MPTP to MPP+ by the DAT. Given the different responses to MPTP treatment of cerebellum and OC found in this study, a plausible hypothesis may be that differences between these two brain areas might be related to the refractoriness of the cerebellum to incorporate the neurotoxin. Thus, we next explored whether the differences in MPTP toxicity in this nerve structures may be related to a distinct distribution of DAT in these mice.

**FIGURE 7:**
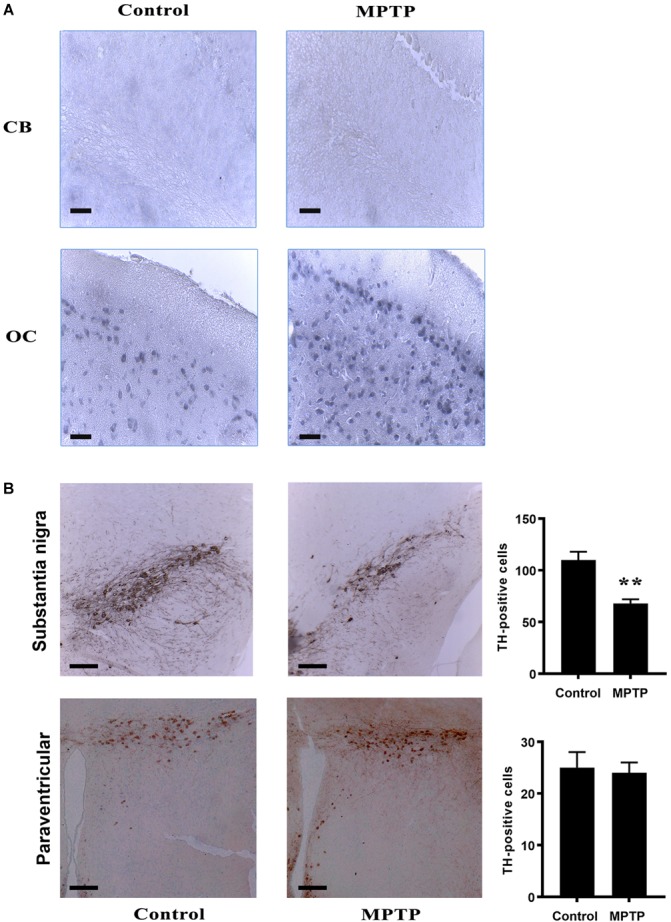
**(A)** Immunohistochemistry analysis of brain and cerebellum sections of the different mice cohorts. **(A)** IHC for DAT in Cerebellum (CB) and occipital cortex (OC) both in control and MPTP treated 14 month-old mice. Notice that, in cerebellum, no DAT positive cells were observed in any of the immunohistochemical sections. In contrast, OC shows DAT immunostaining pattern. **(B)** Tyrosine Hydroxylase IHC in *substantia nigra* and paraventricular nucleus in both control and treated 14 month-old mice. Treatment produced a significant decrease in TH immunoreactivity in *substantia nigra* while no effect was show in paraventricular nucleus. Bar diagrams indicate the relative immunostaining of the different protein markers in 14 month old control and MPTP-treated mice. ^∗∗^ statistically different at *p* < 0.01 4 brains were used for each experimental group.

We performed inmmunohistochemical analyses using anti-DAT antibody in both control and MPTP-treated mice nerve tissues (Figure 7). The results revealed that DAT transporter was only expressed in neurons of the occipital cortex (OC) but not of the cerebellum (CB, Figure 7A). The panel in Figure 7A shows a representation of 14 month-age animals.

Furthermore, we also checked for dopaminergic neurons in these experimental groups, using an anti-TH antibody to stain tissue sections of the same subject. The results indicated the presence of TH-immunoreactive cells in the *substantia nigra*. However, in particular in aged mice, we detected a considerable reduction of TH-positive cells in this region as a result of MPTP treatment (Figure 7B). A similar downward trend was observed in young MPTP-treated animals as compared to controls (data not shown). For comparison, paraventricular regions of aged control and MPTP-treated animals were also used (Figure 7B). In contrast with the *substantia nigra*, a similar amount of TH-positive neurons was quantified in paraventricular region, suggesting that this brain area may not be affected by the neurotoxic treatment.

## Discussion

In the present study, we show that cerebellar LR and non-raft domains exhibit different lipid compositions both at the level of lipid classes and fatty acids in polar lipids. Some lipid classes are highly concentrated exclusively in LR (SM, SULF, and CER). Similarly, higher levels of DHA and AA, 18:2n-6, 18:1n-7 DMA, 18:1n-9 DMA, 20:3n-6 and lower levels of 16:0, 18:1n-7, 22:4n-6, 20:1n-9, 24:1n-9 were observed in non-raft fractions, some of these differences being significant depending on the treatment and age. Overall, LR are more saturated, contain less unsaturated lipid species (in particular for PUFA), and have lower unsaturation and peroxidability indexes compared to NR. These observations are in close agreement with the concept of LR as highly saturated SM -rich membrane microdomains (Brown and London, 2000; Pike, 2006; Allen et al., 2007), and demonstrate the high degree of purity of LR used in this study. We also observed in this work some remarkable changes in distinct ganglioside species (GM1, GD1a, GD1b, and GT1b), which are highly abundant in raft microstructures as part of their intrinsic structure. In this work, we did not observe in cerebellum a significant unbalance of these ganglioside species in raft fractions following MPTP treatment. This is in contrast with previous observations in cortical brain regions, where we observed important anomalies in the levels of GD1a, GD1b, and GT1b species, in particular in aged mice exposed to neurotoxicity (unpublished results). These data indicate that the neurotoxic treatment does not significantly affect ganglioside partitioning in cerebellar micromembrane structures.

To our knowledge, this is the first detailed study showing the differential lipid composition of cerebellar membrane microdomains. The physicochemical impact of this differential composition between rafts and NR is that cell membranes are compartmentalized with raft domains exhibiting closer lipid packing, higher membrane microviscosity and lipid order, lower rotational motion, and restricted lateral movement, than the surrounding non-raft regions (Pike, 2006; Demchenko et al., 2009; Lingwood and Simons, 2010; Simons and Sampaio, 2011; Díaz et al., 2012, 2015).

Aging is known as one of the main factors associated with the development of PD and other neurodegenerative diseases, where profound changes in membrane microdomains have been reported (Martín et al., 2010; Fabelo et al., 2011). However, neuronal membrane alterations in cerebellum have been little studied. In the present study, we found worthwhile analyzing its effects on membrane lipid fractionation from cerebellum as a result of aging and MPTP PD-like neurotoxicity. We observed changes associated with aging for main phospholipids (PC, PS, and PI), with a significant increase in LR of older control animals, which results in a higher phospholipid-to-cholesterol ratio. In NR, the effects of aging were more evident for PE and TPL (which increased in older animals) and CHO (which showed a clear trend to diminish in older control animals). This is in line with previous data indicating that bulk phospholipid levels in human brain (including whole brain, cortex hippocampus, and cerebellum) decrease with aging (Söderberg et al., 1990). Since the expression of *HMG-CoA* gene does not change as a function of aging in control animals, these changes indicate a remodeling of membrane phospholipids and suggest a displacement of CHO from lipid raft to NR as a result of aging, as it has been recently reviewed by Colin et al. (2016). In agreement, we have recently demonstrated in mouse frontal cortex that the reduction in CHO content and the increase in phospholipids and phospholipid/CHO ratio are part of the non-pathological program occurring with the progression of aging (Fabelo et al., 2012). For instance, lipid rearrangements in membrane microenvironments may also affect the behavior of proteins integrated in these structures. A plausible hypothesis may be that subtle early changes in the lipid composition of membrane microstructures may promote abnormal protein aggregations and trafficking, that may ultimately affect protein-lipid microenvironments thereby modifying intracellular responses related to, among others, neurotransmitter, and hormone receptors. Likewise, we have also observed in LR from human frontal cortex a “lipid raft aging” phenomena, and demonstrated that CHO content is negatively correlated with aging, this effect being more evident in women than in men (Díaz et al., 2018).

However, unlike frontal cortex from mice and human where alterations in PUFA and some SAT were reported (Fabelo et al., 2012), fatty acid content was not significantly affected by aging in LR from human cerebellum (Fabelo et al., 2014). In line with this, cerebellar samples from aged and MPTP-treated mice revealed that fatty acids were highly stable in the different cohorts of the present work. This phenomenon suggests the existence of efficient lipostatic mechanisms preserving membrane integrity specifically in cerebellum, even under neurodegenerative conditions (Fabelo et al., 2014; Colin et al., 2016; Marin et al., 2016). Conversely, the results in OC shown here reflect a significant increase in CHO and a reduction of SE contents in older control mice. Interestingly, these results are in agreement with those obtained for gene expression, i.e., expression of *Acat2* is increased and *HMG-CoA* slightly reduced in control older mice. It is worth mentioning that according to EST resources *Acat2* gene is highly expressed in mouse brains^1^ though to lower levels than *Acat1* gene^2^. Further, differences in fatty acids from polar lipids were observed as a result of aging in the OC of control mice. In general, levels of monounsaturated were increased though only statistically significant for 16:1n-7 and 18:1n-7 DMA. Again, these results agree with the expression levels of *Scd* genes (encoding for Δ9 desaturases), which were higher in occipital cortices from older mice.

To further extend the correlation between gene expression involved in lipid metabolism affecting neuronal lipid homeostasis, we have also addressed the effects of aging and MPTP treatment on the lipid composition and lipid-related gene expression in the cerebellum and OC of mice. Considering the fact that the proportion of CHO vs. PUFA is a main parameter of lipid raft stability (Díaz et al., 2016), we selected for our study a group of genes involved, on the one hand, in CHO metabolism (such as *HMG-CoAR*, *Acat1-3*, and *Scd1-2*) and, on the other hand, in the biosynthetic pathway for PUFA (*elovl2-5*, *fads1-2*, *acox1*, and *scd1*). We observed that cerebellum, but not OC, exhibits a high degree of refractoriness to MPTP treatment and that only small changes in lipid composition of microdomains or TPL occurs in response to the neurotoxin. Conversely, in the OC, the effect of MPTP treatment is closely related to aging. Thus, in MPTP-treated animals we found significant increases in *Elovl2*, *Gpx4_m_*_+c_, *Fads2* (*p* < 0.1) and reduction on the expression of *Acat1*, *Acat3*, and *Scd1* (*p* < 0.1) in older mice compared to 6-months-old mice. Further, compared to control animals at the age of 14 months, a number of genes were upregulated, including *Acat2*, *Acox1*, *Elovl2*, *Elovl4*, *Scd1*, and *Scd2* (*p* < 0.1). These results indicate that MPTP treatment modifies the transcriptional program of lipid-related genes involved in biosynthesis of monounsaturated and polyunsaturated fatty acids (reviewed in Guillou et al., 2010).

It is meaningful the fact that *Gpx4* genes were upregulated by two-fold in 6-months mice. The peroxidases encoded by these genes are essential for the homeostasis of membrane lipids, as they are the only enzymes capable to reduce oxidized membrane phospholipids without the need of a phospholipase-2 catalyzed reaction (Imai et al., 2003; Savaskan et al., 2007; Casañas-Sánchez et al., 2015). Thus it seems that upon initial exposure to MPTP, cortical cells in aged animals stimulate the expression *Gpx4* genes to minimize the pro-oxidant effects of MPTP (Zang and Misra, 1993; Gerlach and Riederer, 1996; Maries et al., 2003). However, in MPTP-treated older mice such upregulation was not observed, rendering membrane lipids more prone to oxidative damage. Further, the fact that the expression of *Acat1*, *Acat3*, and *Scd1* genes was increased in older MPTP-treated mice may indicate a modification in the contents of sterol esters and monounsaturated fatty acids. This gene upregulation may compensate the depletion of PUFA and of phospholipid/CHO in membranes as a mechanism to maintain lipid matrix balance. Our last investigations in this issue indicates that this represents a common strategy observed in several neurodegenerative disorders to maintain membrane fluidity (Díaz et al., 2012, 2015; Fabelo et al., 2014).

Furthermore, compared to control tissues, older MPTP-treated animals exhibited significantly lower expression of genes encoding for elongases (*Elovl2, Elovl4*, and *Elovl5*), *Acox1* (involved in the final step of n-3 and n-6 PUFA synthesis), and *Scd1* and *Scd2* (responsible for synthesis of monounsaturated fatty acids from their saturated precursors), which lead us to surmise that MPTP dramatically impacts physicochemical properties of nerve cell membranes. To our knowledge, these results represent the first demonstration that MPTP modulates lipid-related gene expression in brain tissues. Beyond the demonstration that cerebellar and OC cells are endowed with the machinery for the biosynthesis of PUFA, sterol esters and CHO, our data altogether demonstrate that MPTP treatment modifies the transcriptional expression of many of these genes, but only in the OC.

The striking difference in the response to MPTP between cerebellar and OC led us to explore the plausible link between the selective effects of the neurotoxin and the immunohistochemical features of their cellular populations.

Our present results point to a crucial role of DAT in MPTP-induced toxicity. We show here that, unlike in the OC, DAT is not expressed in cerebellum, even though TH-immunoreactive cells (dopaminergic) are present in this brain region. On its own, this observation may explain why cerebellar cells are resistant to MPTP treatment, as the oxidized metabolite MPP^+^ must enter dopaminergic cells through DAT transporter. Once in the cell, MPP+ inhibits the mitochondrial complex I and leads to ROS generation and cell death (Maries et al., 2003; Meredith and Rademacher, 2011). In agreement, in a search in gene expression databases, we have found that DAT-encoding gene in mouse, *Slc6a3*, is expressed at very low or negligible levels in cerebellum^3^. Similar outcomes were obtained when explored in human expression databases^4^.

However, a main limitation of this study is that PD neuropathology is not only influenced by dopaminergic degeneration. For instance, the existence of compensatory mechanisms in the early pre-symptomatic phase of the disease as well as during the clinical progression has been reported. These mechanisms may be molecular and cellular dopaminergic but also non-dopaminergic events, in which the cerebellum may also be involved (Blesa et al., 2017). Indeed, motor features in the onset of PD has been associated with the recruitment of the cerebellum-thalamo-cortical circuit (Wu and Hallett, 2013), although it remains partially uncharacterized.

In conclusion, we demonstrate here that aging is accompanied by subtle changes in nerve membrane lipid composition both in cerebellum and OC. However, MPTP treatment leads to important changes in lipid signature of OC which are not observed in the cerebellum. In the OC, the effects of this neurotoxin are facilitated by aging and involve not only alterations in the lipid contents but also in the expression of lipid biosynthesis related genes. These effects are likely associated to the differential expression of DAT in cerebellar and OC nerve cell populations.

Further studies will be required to characterize the distinct lipid-driven regulation of membrane integrity associated with neuronal preservation against PD pathogenesis.

## Ethics Statement

Animal Facilities Service and Ethics Comitee from University of La Laguna (Spain). All procedures were reviewed and approved by the Ethics Committee at the Universidad de La Laguna.

## Author Contributions

MD designed the study and wrote the manuscript. JP and VC-S performed the genetic experiments. AL-A and RM performed the immunohistochemical studies. DB performed the lipid analyses.

## Conflict of Interest Statement

The authors declare that the research was conducted in the absence of any commercial or financial relationships that could be construed as a potential conflict of interest.

## Footnotes

1 https://www.ncbi.nlm.nih.gov/UniGene/ESTProfileViewer.cgi?uglist=Mm.439711

2 https://www.ncbi.nlm.nih.gov/UniGene/ESTProfileViewer.cgi?uglist=Mm.293233

3 https://www.ncbi.nlm.nih.gov/gene/13162

4 https://www.ncbi.nlm.nih.gov/gene/6531
